# Magnetic Nanozyme Based on Loading Nitrogen-Doped Carbon Dots on Mesoporous Fe_3_O_4_ Nanoparticles for the Colorimetric Detection of Glucose

**DOI:** 10.3390/molecules28124573

**Published:** 2023-06-06

**Authors:** Yunxi Huang, Zhanling Ding, Yutong Li, Fengna Xi, Junjie Liu

**Affiliations:** 1 Department of Medical Ultrasound, Guangxi Medical University Cancer Hospital, Guangxi Medical University, Nanning 530021, China; huangyunxi@stu.gxmu.edu.cn (Y.H.); h1991001@sr.gxmu.edu.cn (Z.D.); 2 Department of Chemistry, Zhejiang Sci-Tech University, Hangzhou 310018, China; 202030107261@mails.zstu.edu.cn

**Keywords:** colorimetric detection, magnetic nanozyme, nitrogen-doped carbon dots, mesoporous Fe_3_O_4_ nanoparticle, glucose

## Abstract

The simple and accurate monitoring of blood glucose level is of great significance for the prevention and control of diabetes. In this work, a magnetic nanozyme was fabricated based on loading nitrogen-doped carbon dots (N-CDs) on mesoporous Fe_3_O_4_ nanoparticles for the colorimetric detection of glucose in human serum. Mesoporous Fe_3_O_4_ nanoparticles were easily synthesized using a solvothermal method, and N-CDs were then prepared in situ and loaded on the Fe_3_O_4_ nanoparticles, leading to a magnetic N-CDs/Fe_3_O_4_ nanocomposite. The N-CDs/Fe_3_O_4_ nanocomposite exhibited good peroxidase-like activity and could catalyze the oxidation of the colorless enzyme substrate 3,3′,5,5′-tetramethylbenzidine (TMB) to blue TMB oxide (ox-TMB) in the presence of hydrogen peroxide (H_2_O_2_). When the N-CDs/Fe_3_O_4_ nanozyme was combined with glucose oxidase (Gox), Gox catalyzed the oxidization of glucose, producing H_2_O_2_ and leading to the oxidation of TMB under the catalysis of the N-CDs/Fe_3_O_4_ nanozyme. Based on this mechanism, a colorimetric sensor was constructed for the sensitive detection of glucose. The linear range for glucose detection was from 1 to 180 μM, and the limit of detection (LOD) was 0.56 μM. The recovered nanozyme through magnetic separation showed good reusability. The visual detection of glucose was also realized by preparing an integrated agarose hydrogel containing the N-CDs/Fe_3_O_4_ nanozyme, glucose oxidase, and TMB. The colorimetric detection platform has an enormous potential for the convenient detection of metabolites.

## 1. Introduction

Diabetes is a metabolic disease characterized by an elevated glucose (Glu) concentration in the blood, which affects millions of people around the world [1]. A long-term high blood glucose may lead to organ failure and tissue damage, causing various complications such as blindness, cardiovascular disease, and kidney failure [2]. Therefore, the accurate and timely monitoring of blood glucose level is of great significance for the prevention and control of diabetes. With the development of clinical testing, daily home-based health monitoring, and personalized treatment, the convenient glucose detection has attracted widespread attention. On the one hand, glucose detection using body fluid samples such as sweat, skin interstitial fluid, tears, saliva, and urine as substitutes for blood is widely researched. On the other hand, methods such as visual, continuous, non-invasive, or wearable device-based glucose monitoring is constantly investigated. Until now, various technologies have been developed for the detection of glucose, such as electrochemical sensors, colorimetric methods, fluorescence methods, and chemiluminescence methods [3,4,5,6]. Amongst them, colorimetric detection has the advantages of requiring a simple equipment, having a low experimental cost and high sensitivity, and being easy to combine with smartphones to achieve visual detection. The simple and convenient colorimetric detection and visual glucose detection are highly desirable.

In a typical colorimetric method, glucose is oxidized by glucose oxidase to produce gluconic acid and H_2_O_2_ [7]. Then, the concentration of glucose could be detected by measuring the changes in the peroxidase substrates from colorless to colored products in the presence of peroxidase [8]. However, the natural peroxidase catalyst still has some drawbacks. Commonly, natural enzymes and their activities are sensitive to the external environment including temperature and pH value. In addition, natural enzymes are also expensive and difficult to be recycled. Substitutes for natural peroxidase have been paid great attention. Nanozymes are nanomaterials with catalytic activity similar to that of natural enzymes [9,10]. Owing to the advantages of high stability in harsh environments, ease of large-scale production, and low cost, nanozymes have been widely used in various fields such as biosensing, pollutant degradation, antibacterial material production, biomedical applications, etc. [10,11,12]. The development of novel and highly active nanozymes for the detection of glucose concentration in the blood is of great significance.

Magnetic nanomaterials are easy to be separated and recycled. Iron oxides have attracted much attention due to their strong magnetic responsiveness and biocompatibility [13]. Especially, Fe_3_O_4_ nanomaterials play an important role in the biological or medical fields due to their excellent magnetic responsiveness and catalytic properties [14]. It is proven that Fe_3_O_4_ nanomaterials have inherent peroxidase activity and can catalyze the coloration of different peroxidase substrates in the presence of H_2_O_2_, such as 3,3′,5,5′-tetramethylbenzidine (TMB), diazoaminobenzene (DAB), phenylenediamine (OPD), 2,2′-azo bis (3-ethylbenzothiazole-6-sulfonic acid) diamine salt (ABTS), etc. [15]. However, the inherent nanozyme activity of Fe_3_O_4_ nanomaterials is not high. The preparation of composite materials using Fe_3_O_4_ as the supporting matrix to obtain magnetic nanozymes with high catalytic activity is highly desirable.

Nanocarbon materials have widely adjustable structures, excellent electrical or optical properties, high stability, and biocompatibility, exhibiting great potential in chemical sensing [16,17], energy monitoring [18], catalysis [19], etc. In addition to three-dimensional (3D) and two-dimensional (2D) carbon nanomaterials [20,21,22], zero-dimensional (0D) carbon nanomaterials including graphene quantum dots (GQDs) and carbon dots (CDs) have received widespread attention in recent years [18,23]. CDs usually have unique optical properties, (e.g., fluorescence or electrochemiluminescence) properties, good water solubility, low toxicity, environmental friendliness, low cost, and good biocompatibility [24,25,26,27]. Up to now, many synthesis methods have been developed for the synthesis of CDs including arc discharge, laser ablation, electrochemical synthesis, chemical oxidation, hydrothermal synthesis, microwave synthesis, etc. [28,29,30]. Very recently, researchers proved that functional carbon dots with high nanozyme activity can be obtained by changing the structure of the carbon dots [31,32,33,34]. For example, nitrogen doping can significantly increase the pseudo-peroxidase activity of carbon nanomaterials [9,11,12]. Magnetic nanozymes with high activity are expected to be obtained by modifying Fe_3_O_4_ nanomaterials with CD nanozymes.

In this work, magnetic nanozymes with a high pseudo-peroxidase activity were prepared, and a colorimetric platform for glucose detection with high sensitivity was constructed. Firstly, mesoporous Fe_3_O_4_ nanoparticles were synthesized. Subsequently, nitrogen-doped carbon dots (N-CDs) were in situ synthesized and loaded on mesoporous Fe_3_O_4_ nanoparticles to obtain a magnetic nanocomposite (N-CDs/Fe_3_O_4_). N-CDs/Fe_3_O_4_ exhibited peroxidase-like activity and could be recovered through magnetic separation. A colorimetric sensor was constructed for the detection of glucose based on the good peroxidase activity of N-CDs/Fe_3_O_4_. The visual detection of glucose was also investigated by preparing an integrated agarose hydrogel containing the N-CDs/Fe_3_O_4_ nanozyme, glucose oxidase, and TMB. The developed magnetic nanozyme has the advantages of simple synthesis, low cost, high catalytic ability, and good reusability, indicating its great potential for the convenient detection of glucose.

## 2. Results and Discussion

### 2.1. Strategy for the Colorimetric Detection of Glucose Based on the N-CDs/Fe_3_O_4_ Magnetic Nanozyme

**Figure 1:**
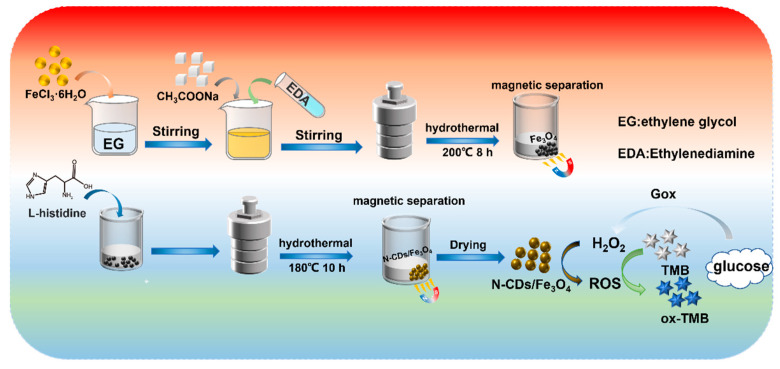
Schematic illustration of the synthesis of the magnetic N-CDs/Fe_3_O_4_ nanozyme and of the colorimetric detection of glucose by combining the N-CDs/Fe_3_O_4_ nanozyme with glucose oxidase.

Mesoporous materials have high specific surface area, regular and ordered pore structure, narrow pore size distribution, and continuously adjustable pore size, which play an important role in adsorption, separation, catalytic reactions, and other applications [35,36,37,38]. As illustrated in Figure 1, mesoporous Fe_3_O_4_ nanomaterial was firstly synthesized using a solvothermal method [39]. Subsequently, nitrogen-doped carbon dots (N-CDs) with peroxidase-like activity were synthesized in situ using a hydrothermal method in the presence of the Fe_3_O_4_ nanomaterial. A common nitrogen-containing amino acid, histidine, was used as the carbon source for the synthesis of N-CDs. The as-prepared N-CDs/Fe_3_O_4_ nanocomposite has the advantages of a high peroxidase-like activity, magnetic separation ability, simple preparation, and low cost. By combining the production of H_2_O_2_ by the glucose oxidase (Gox)-catalyzed oxidation of glucose, the colorimetric detection of glucose could be achieved.

### 2.2. Characterization of N-CDs/Fe_3_O_4_ Magnetic Nanozyme

**Figure 2:**
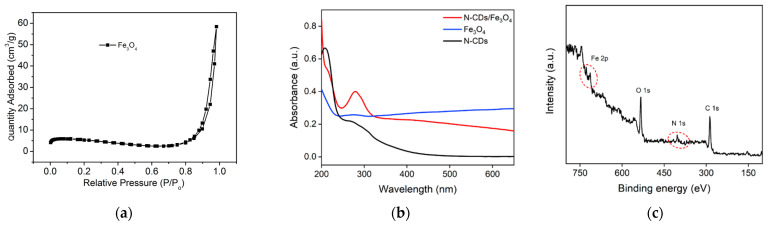
(**a**) N_2_ adsorption–desorption isotherm of the Fe_3_O_4_ nanomaterial. (**b**) UV–Vis absorption spectra of N-CDs, Fe_3_O_4_, and N-CDs/Fe_3_O_4_. (**c**) XPS survey spectrum of N-CDs/Fe_3_O_4_.

The N_2_ adsorption–desorption technology was used to explore the pore structure of the produced Fe_3_O_4_ nanomaterials. Figure 2a shows the N_2_ adsorption–desorption isotherm of the Fe_3_O_4_ nanomaterial. As seen, the isotherm was a typical type IV isotherm with an H3 hysteresis loop, indicating the mesoporous nature of the Fe_3_O_4_ nanomaterial [40]. Based on the BJH method, two types of mesoporous structures were revealed. One, with an average diameter of 6.5 nm, corresponded to pores formed between the stacked Fe_3_O_4_ nanoparticles. The other, with an average diameter of 7 nm, indicated the mesopores on the surface of the nanoparticles. Thus, the as-synthesized Fe_3_O_4_ is a mesoporous nanomaterial.

The N-CDs/Fe_3_O_4_ nanocomposite was characterized by UV–visible absorption spectroscopy. As shown in Figure 2b, the absorption curve of mesoporous Fe_3_O_4_ exhibited a weak absorption peak at around 280 nm, which was attributed to the n-π* transition of the C=O bond in the precursor of sodium acetate. The spectra of N-CDs, showed the characteristic absorption peaks at ~220 nm and 280 nm resulting from the π-π* transition of the C=C bond and the n-π* transition of the C=O bond, respectively. The N-CDs/Fe_3_O_4_ nanocomposite exhibited the characteristic absorption peaks of both Fe_3_O_4_ and N-CDs, indicating the effective preparation of the nanocomposite. In addition, the absorption peak strength of the N-CDs/Fe_3_O_4_ nanocomposite at 280 nm was significantly higher than that of N-CDs and Fe_3_O_4_ at 280 nm. This might be due to the enrichment of N-CDs on the surface of the Fe_3_O_4_ nanoparticles.

**Figure 3:**
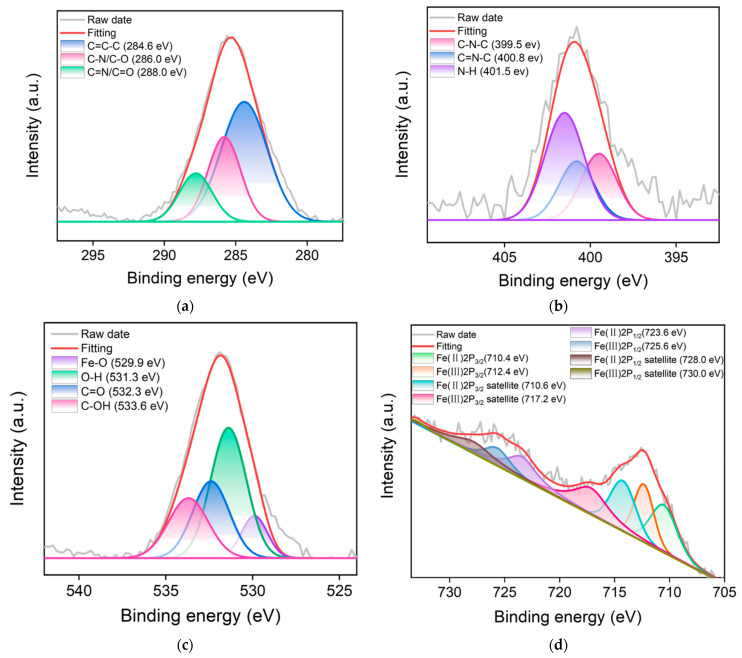
High-resolution XPS C1s (**a**), N1s (**b**), O1s (**c**), Fe2p (**d**) spectra of N-CDs/Fe_3_O_4_.

The elemental and chemical groups on the surface of N-CDs/Fe_3_O_4_ were characterized by X-ray photoelectron spectroscopy (XPS). Figure 2c shows the XPS survey spectrum of N-CDs/Fe_3_O_4_, which revealed four elements including Fe, C, N, and O with the corresponding element contents of 1.8%, 64.8%, 7.9%, and 25.5%. ICP-OES was also applied to accurately analyze the Fe content in N-CDs/Fe_3_O_4_. The mass fraction ratio of Fe was 61.4%. This indicated that the surface of mesoporous Fe_3_O_4_ was enveloped by N-CDs. Thus, the iron content in the entire nanocomposite determined by ICP-OES was remarkably higher than the iron content on the surface determined by XPS. Figure 3a shows the high-resolution C1s spectrum of N-CDs/Fe_3_O_4_. The peaks with binding energy of 284.6 eV, 286.0 eV, and 288.0 eV correspond to the structure of graphite C (C-C=C, sp^2^ carbon), the C-N/C-O bond, or the C=N/C=O bond. The corresponding high-resolution N1s spectrum in Figure 3b displays peaks with binding energy of 399.5 eV, 400.8 eV, and 401.5 eV, corresponding to the C-N-C bond, the C=N-C bond, and the N-H bond, respectively. Figure 3c shows the high-resolution O1s spectrum of N-CDs/Fe_3_O_4_. The peaks at 529.9 eV, 531.3 eV, 532.3 eV, and 533.6 eV are related to the Fe-O bond, the O-H bond, the C=O, and the C-OH bond, respectively. The characteristic peaks in the high-resolution Fe2p spectrum of N-CDs/Fe_3_O_4_ confirmed the presence of Fe^2+^ and Fe^3+^ in N-CDs/Fe_3_O_4_ (Figure 3d) [41,42]. The above results indicated that N-CDs were successfully modified on the surface of the mesoporous Fe_3_O_4_ nanomaterial, demonstrating the successful formation of the nanocomposite.

**Figure 4:**
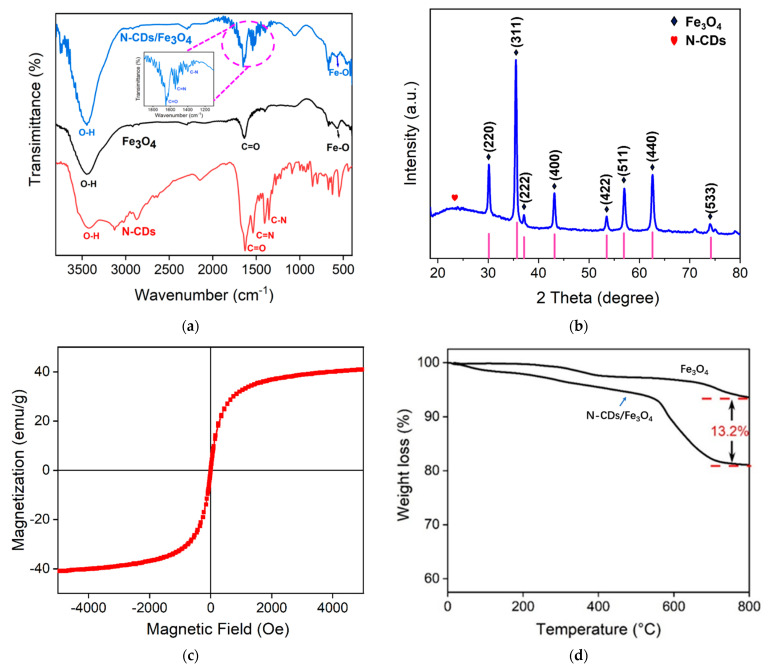
(**a**) FT-IR spectra of N-CDs, Fe_3_O_4_, and N-CDs/Fe_3_O_4_. (**b**) XRD diffraction spectra of N-CDs/Fe_3_O_4_. (**c**) Hysteresis loop of N-CDs/Fe_3_O_4_. (**d**) Thermogravimetric curves of N-CDs/Fe_3_O_4_ and Fe_3_O_4_.

The functional groups of N-CDs/Fe_3_O_4_ were also characterized by Fourier transform infrared spectroscopy (FT-IR). As shown in Figure 4a, N-CDs/Fe_3_O_4_ exhibited the characteristic peaks of both N-CDs and Fe_3_O_4_. The absorption peak at around 3400 cm^−1^ was attributed to the stretching vibration of O-H, and the peak at 1628 cm^−1^ was attributed to the stretching vibration of C=O. The peaks at 1534 cm^−1^ and 1357 cm^−1^ resulted from the stretching vibration of C=N and C-N, respectively. The absorption peak around 580 cm^−1^ indicated the stretching vibration of Fe-O. The C=O stretching might be attributed to the residual acetate group on the surface of the Fe_3_O_4_ nanoparticles, which derived from the raw material acetate. The presence of these characteristic groups proved the successful preparation of the N-CDs/Fe_3_O_4_ nanocomposite. In addition, the abundant oxygen-containing and nitrogen-containing functional groups on N-CDs/Fe_3_O_4_ will favor the good dispersion of the magnetic nanocomposite in water.

The crystal structure of N-CDs/Fe_3_O_4_ was characterized by X-ray diffraction (XRD). The results are displayed in Figure 4b. The wide peak at 24° might be caused by the amorphous carbon of N-CDs. The peaks at 30.1°, 35.7°, 43.1°, 53.5°, 56.9°, 62.6°, and 74.2° correspond to the (220), (311), (400), (422), (511), (440), and (533) planes of Fe_3_O_4_, respectively [39,42,43]. This phenomenon indicated that the modification of the Fe_3_O_4_ nanocomposite by N-CDs did not affect the inherent crystal structure of Fe_3_O_4_.

**Figure 5:**
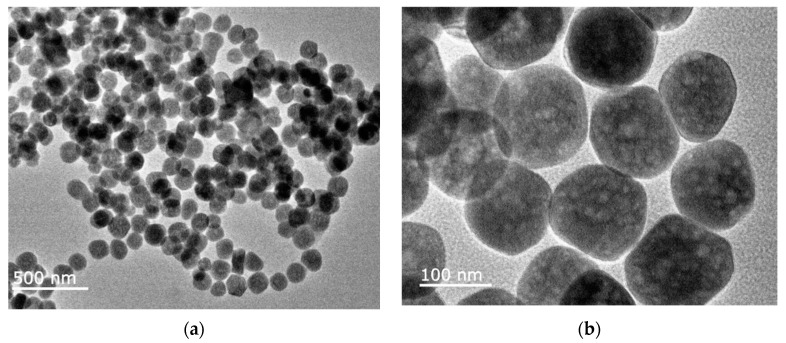
TEM images of N-CDs/Fe_3_O_4_ at low (**a**) and high (**b**) magnifications.

The ferromagnetic properties of N-CDs/Fe_3_O_4_ were characterized by measuring the hysteresis loop. As shown in Figure 4c, the saturation magnetization of N-CDs/Fe_3_O_4_ was 40.9 emu/g, indicating the magnetic properties of the N-CDs/Fe_3_O_4_ nanocomposite. The mass fraction of N-CDs in N-CDs/Fe_3_O_4_ was characterized by thermogravimetric analysis. As shown in Figure 4d, N-CDs/Fe_3_O_4_ exhibited good thermal stability below 550 °C. When the temperature changed from 550 °C to 800 °C, the mass of N-CDs/Fe_3_O_4_ decreased sharply to maintain an equilibrium. In comparison with Fe_3_O_4_, a mass ratio of 13.2% was obtained resulting from the thermal decomposition of N-CDs. The morphology and size of N-CDs/Fe_3_O_4_ were characterized by transmission electron microscopy (TEM). As shown in Figure 5, N-CDs/Fe_3_O_4_ appeared spherical in shape, with a particle size of ~130 nm.

### 2.3. Peroxide-Like Activity of N-CDs/Fe_3_O_4_

**Figure 6:**
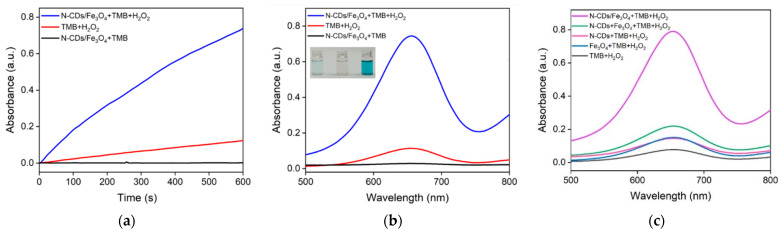
(**a**) Time-dependent change of the absorbance at 652 nm and (**b**) absorbance spectra of different mixed solutions of N-CDs/Fe_3_O_4_, H_2_O_2_, and TMB after 10 min of reaction. Inset in (**b**) shows photographs of the TMB solution in the presence of H_2_O_2_ (**left**), N-CDs/Fe_3_O_4_ (**middle**). and N-CDs/Fe_3_O_4_ + H_2_O_2_ (**right**). (**c**) Absorbance spectra of different mixture solutions (as indicated) after 10 min of reaction. The concentrations of TMB, N-CDs, Fe_3_O_4_, N-CDs/Fe_3_O_4_, and H_2_O_2_ were 0.5 mM, 1.3 μg/mL, 8.7 μg/mL, 10 μg/mL, and 6.6 mM, respectively.

The peroxidase-like activity of N-CDs/Fe_3_O_4_ was investigated by catalyzing the oxidation of TMB by H_2_O_2_. Figure 6a shows the absorbance values of different solutions at 652 nm when the reaction time changed. As seen, the absorbance value of the solution remained almost unchanged when only N-CDs/Fe_3_O_4_ and TMB were present in the solution, indicating no reaction between N-CDs/Fe_3_O_4_ and TMB. When TMB was mixed with H_2_O_2_, a slight increase in the absorbance of the solution was observed due to the slow oxidation of TMB by H_2_O_2_. When N-CDs/Fe_3_O_4_ was mixed with TMB and H_2_O_2_, the absorbance of the solution significantly increased. After 10 min of reaction, the absorption spectrum of the solution in Figure 6b also confirmed this phenomenon, proving that N-CDs/Fe_3_O_4_ can catalyze the oxidation of TMB by H_2_O_2_ to produce a blue TMB oxide (ox-TMB). 

To investigate the possible synergistic effect of N-CDs and the Fe_3_O_4_ nanoparticles, the catalytic activity of the N-CDs and Fe_3_O_4_ nanoparticles was investigated separately. To keep up with the content of N-CDs and Fe_3_O_4_ nanoparticles in the nanocomposite, the concentration of N-CDs and Fe_3_O_4_ nanoparticles was 13.2% and 86.8% of that of the nanocomposite, respectively. As shown in Figure 6c, when N-CDs and the Fe_3_O_4_ nanoparticles were present alone, they could catalyze the oxidation of TMB by H_2_O_2_, but the absorbance of the reaction solution at 652 nm was not high, indicating low nanozyme activity. It has been proven that the introduction of an N atom to the graphitic carbon structures of CDs can enhance the peroxidase-like activity owing to the enhanced electrical properties and affinity towards substrates [16,23,26]. At the same time, the catalytic activity of Fe_3_O_4_ originates from Fe^2+^ on the surface of the nanoparticles, which undergoes Fenton or Fenton-like reactions, resulting in the oxidation of TMB by H_2_O_2_ [44]. Even if the two were mixed, the absorbance of the solution after reaction was significantly lower than that of the solution obtained using the N-CDs/Fe_3_O_4_ nanocomposite. This phenomenon proved the coordinated effect of N-CDs and Fe_3_O_4_ nanoparticles combined in a nanocomposite, leading to a significant improvement in the catalytic ability. This might be attributed to the spatial confinement of N-CDs by the mesoporous structure of the Fe_3_O_4_ nanocomposite.

**Figure 7:**
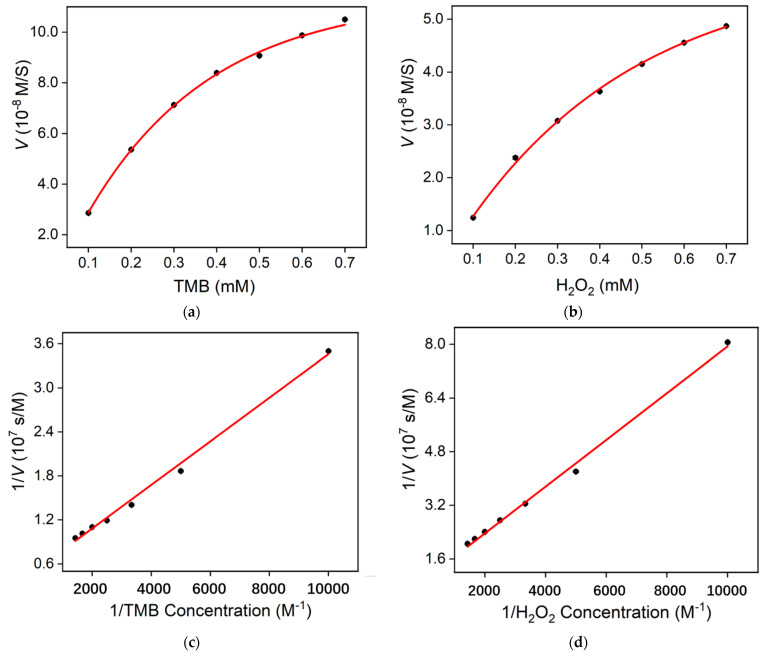
(**a**,**b**) Steady-state kinetic assay of N-CDs/Fe_3_O_4_, where the reaction velocity was determined through the oxidation of TMB based on the absorption at 652 nm with varying concentrations of (**a**) TMB or (**b**) H_2_O_2_. (**c**,**d**) Double-reciprocal plots of N-CDs/Fe_3_O_4_ activity obtained using the Michaelis–Menten model versus various concentrations of TMB (**c**) or H_2_O_2_ (**d**).

The Michaelis constant (*K*m) and maximum reaction rate constant (*V*max) are important parameters to evaluate the activity of nanozymes. Hydrogen peroxide and TMB were applied as substrates to measure the *K*m and *V*max of the nanozyme using the Lineweaver–Burk curves (Figure 7). The *K*m using TMB as the substrate was 0.607 mM, and the *V*max was 2.054 × 10^−7^ M/s (Figure 7a,c). The *K*m value obtained using H_2_O_2_ as the substrate was 0.719 mM, and the *V*max was 1.032 × 10^−8^ M/s (Figure 7b,d).

Commonly, a high *V*max suggests high catalytic activity, whereas a small *K*m indicates a high affinity between enzymes and substrates. Table 1 reports the comparison between *K*m and *V*max of different nanozymes [44,45,46,47,48]. As shown, the *K*m obtained using the developed N-CDs/Fe_3_O_4_ was lower than that obtained with the natural enzyme horseradish peroxidase (HRP) [44] when H_2_O_2_ was applied as the substrate. The *K*m was also lower than that obtained using N-CD [46], Fe_3_O_4_ [44], or the Fe-doped carbon-dot (Fe-CD) [48] nanozyme, but higher than that obtained using a metal oxide hybrid with nitrogen-doped carbon dots [45] or Fe- and N-co-doped CDs (Fe, N-CDs) [47]. In addition, the *V*max was the highest with TMB as the substrate.

Under optimal reaction conditions, the required amount of nanozyme was 1U when 1 μmol of the substrate was used and converted into the product. The specific activity (U/mg) refers to the number of activity units per unit mass of nanozyme. The specific activity of N-CDs/Fe_3_O_4_ was 13.1 U/mg, which was remarkably higher than that of Fe_3_O_4_ (0.215 U/mg). Thus, the modification of mesoporous Fe_3_O_4_ using N-CDs could significantly enhance the nanozyme activity.

### 2.4. Optimization of the Detection Conditions

**Figure 8:**
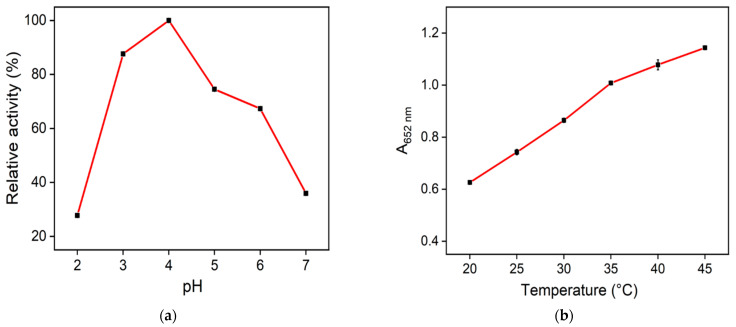
Relative nanozyme activity of N-CDs/Fe_3_O_4_ at different pH (**a**) or temperature (**b**) values.

To obtain the optimal nanozyme reaction conditions, the reaction temperature and pH were optimized. As shown in Figure 8a, the absorbance value of the mixed solution (nanozyme + TMB + H_2_O_2_) at 652 nm reached its maximum at pH 4, indicating that N-CDs/Fe_3_O_4_ had the best peroxidase-like activity in this condition. The absorbance value increased when the reaction increased from 20 °C to 45 °C (Figure 8b). Although the absorbance of the mixture at 45 °C was greater than at 40 °C, the increase was not remarkably high. Considering that 40 °C is close to the physiological temperature, it was chosen for the subsequent experiments. 

### 2.5. Colorimetric Detection of Glucose

Glucose is the main energy source of the human body. Long-term hyperglycemia is likely to lead to the occurrence of diabetes, increasing the risk of heart disease, stroke, blindness, renal failure, peripheral neuropathy, etc. Thus, the effective monitoring of blood glucose is of great significance for the diagnosis of hyperglycemia. In this work, Gox was applied to catalyze the oxidation of glucose to form gluconic acid and hydrogen peroxide. At the same time, H_2_O_2_ was decomposed into reactive oxygen species (ROS) under the catalysis of the peroxidase-like enzyme N-CDs/Fe_3_O_4_ which oxidized the colorless TMB to the blue ox-TMB. The reaction process was monitored by measuring the absorption of the mixture containing Gox, nanozyme, TMB, H_2_O_2_, and different concentrations of glucose.

**Figure 9:**
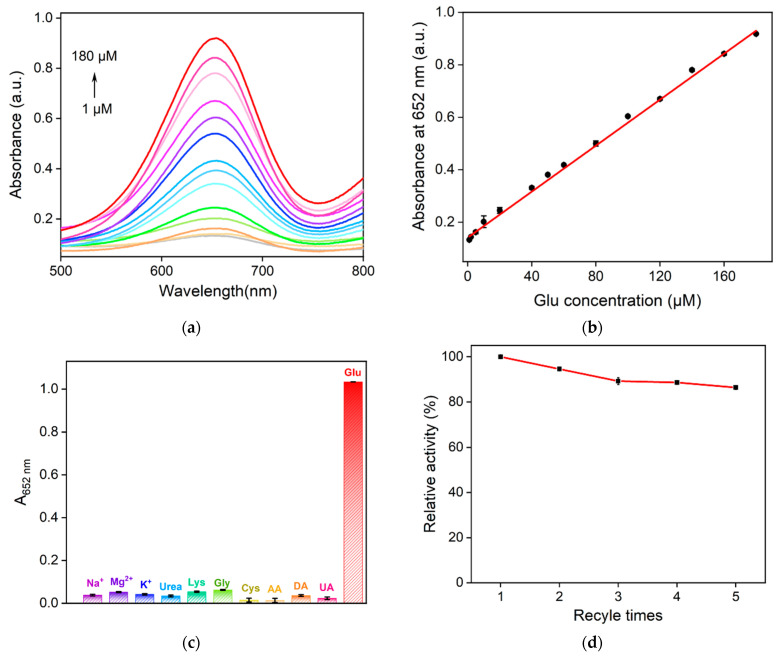
(**a**) Absorbance spectra obtained for the mixture of N-CDs/Fe_3_O_4_ + H_2_O_2_ + TMB in the presence of different concentrations of glucose. (**b**) The corresponding linear calibration plot for the colorimetric detection of glucose. (**c**) The selectivity of the fabricated sensor. (**d**) The reusability of the N-CDs/Fe_3_O_4_ nanozyme.

As shown in Figure 9a, the absorbance of the mixed solution increased with the increase in glucose concentration. The absorbance value of the system at 652 nm was consistent with the glucose (Glu) concentration between 1 and 180 μM (Figure 9b), with a good linear relationship (A = 0.00438 *C* + 0.142, *R*^2^ = 0.997). The limit of detection (LOD) calculated based on three signal-to-noise (*S/N*) values was 0.56 μM. The LOD was lower than that obtained using fluorescent detection based on a CDs/Ag nanoparticle composite (CDs/AgNPs) [49], a GQD/Au nanoparticle composite (GQD/AuNPs) [50], Mg- and N-co-doped CD (Mg,N-CD) [51], and MFNCDs [44] or colorimetric detection using Fe single-site nanozyme (Fe SSN) [52] or Au-Pt nanoclusters (Au-PtNCs) [53], but higher than that obtained using fluorescent detection based on C-dots/Fe^2+^/thiamine (C-dots/Fe^2+^/VB_1_) [54] or colorimetric detection using MFNCDs [44]. In addition, three batches of N-CDs/Fe_3_O_4_ were synthesized independently. The relative standard deviation (RSD) for the determination of glucose (50 μM) was 3.7%, indicating good reproducibility. 

### 2.6. Selectivity of the Sensor and Real Sample Analysis

Selectivity is an important parameter for evaluating a sensor performance. To explore the selectivity of the constructed colorimetric sensor, several common substances in the serum were selected as possible interferences including uric acid (UA), urea, cysteine (Cys), glycine (Gly), lysine (Lys), ascorbic acid (AA), dopamine (DA), Mg^2+^, Na^+^, and K^+^. As shown in Figure 9c, no significant changes in the absorbance at 652 nm was observed even when the concentration of each of the above substance was five times higher than that of glucose, indicating the constructed sensor has good selectivity for the colorimetric detection glucose.

To investigate the application of the constructed sensor in a real application, the glucose level in a healthy woman was determined. The detected glucose concentration (4.89 mM) was quite similar to that obtained using an automatic biochemical analyzer (Ci-8200, Abbott, Washington, DC, USA), indicating good accuracy.

### 2.7. Reusability of N-CDs/Fe_3_O_4_

Because N-CDs/Fe_3_O_4_ has high magnetic susceptibility, it can also be recovered by magnetic separation. The performance of the recovered N-CDs/Fe_3_O_4_ in glucose detection is shown in Figure 9d. As seen, N-CDs/Fe_3_O_4_ still maintained approximately 80% of the original catalytic activity after five cycles of use. Therefore, N-CDs/Fe_3_O_4_ can be recycled and has high reusability.

### 2.8. Visual Glucose Detection Based on an Integrated Hydrogel

**Figure 10:**
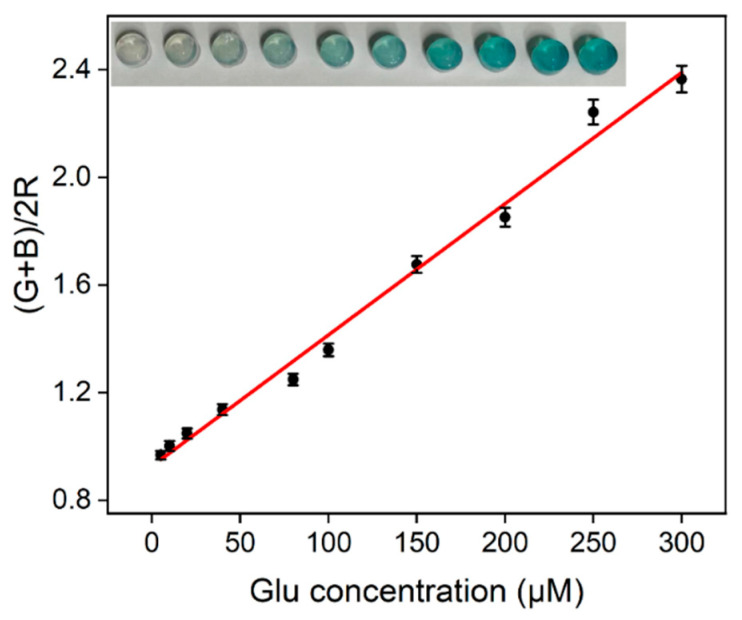
Linear relationship curve between (*G* + *B*)/2*R* of the hydrogel and glucose concentration. Inset shows hydrogel photos taken after the reaction with different concentrations of glucose (the concentration increases from left to right as indicated in the linear regression curve).

Visual detection has the advantages of being fast and providing intuitive results, demonstrating great potential in glucose detection. To explore the possibility of using the nanozyme in visual glucose detection, the N-CDs/Fe_3_O_4_ nanozyme and natural glucose oxidase were integrated into an agarose gel. Multienzyme-mediated catalysis in a hydrogel can improve the catalytic efficiency because of the limited distribution in space and the relative high concentration of the substrates. More importantly, the detection operation could be greatly simplified since the identification unit and the signal module are all integrated in the hydrogel. When the integrated hydrogel reacted with glucose at different concentrations, photos were taken with a smartphone, and the red (*R*), green (*G*), and blue (*B*) optical primary colors were extracted. As shown in Figure 10, a good linear relationship between (*G* + *B*)/2*R* (y) and Glu concentration (*C*) was observed when the concentration of glucose ranged from 5 μM to 300 μM (y = 0.0048*C* + 0.93, *R*^2^ = 0.991). The detection limit was 2.8 μM. 

## 3. Materials and Methods

### 3.1. Chemicals and Materials

Sodium acetate, 1,2-ethylenediamine, FeCl_3_·6H_2_O, L-histidine, 3,3′,5,5′-tetramethylbenzidine, hydrogen peroxide, urea, potassium bromide, magnesium sulfate, cysteine, glycine, glutamate, ascorbic acid, glucose, and glucose oxidase were purchased from Aladdin Biochemical Technology Co., Ltd. (Shanghai, China). Sodium chloride (NaCl) and urea (Urea) were purchased from Tianjin Yongda Chemical Reagent Co., Ltd. (Tianjing, China). Ethylene glycol was purchased from McLean Biochemical Technology Co., Ltd. (Shanghai, China). All reagents were analytically pure and were not further purified before use. Ultrapure water (18 MΩ·cm) was used in the experiments.

### 3.2. Characteriaztions and Instrumentations

The fluorescence excitation and emission spectra of N-CDs were measured using a fluorescence spectrometer (LuoroMax-4, Horiba, France). The size and morphology of N-CDs were characterized using transmission electron microscopy (TEM, JEM-2100, Japan Electronics Corporation, Tokyo, Japan). The chemical groups of the nanomaterials were determined by X-ray photoelectron spectroscopy (XPS, PHI5300, PE company, Boston, MA, USA). Fourier transform infrared spectrometry (Vertex 70, Bruker company in Bremen, Germany) was applied to characterize the chemical groups of the synthesized nanomaterials. The crystal structure of N-CDs/Fe_3_O_4_ was characterized using a powder diffractometer (Bruker, Germany). The UV–visible absorption spectrum was determined using a UV-2450 spectrophotometer (UV-Vis, Shimadzu Corporation, Tokyo, Japan). The content of Fe in N-CDs/Fe_3_O_4_ was measured by an Agilent 730 inductively coupled plasma emission spectrometer (ICP-OES, Agilent Corporation, Palo Alto, CA, USA). The mass composition of the N-CDs/Fe_3_O_4_ components was characterized by scanning calorimetry and thermogravimetric synchrotron diffraction (TGA/DSC, Mettler Toledo, Zurich, Switzerland). The N_2_ adsorption/desorption isotherm was obtained at 77 K using the ASAP2020 physical adsorption instrument. Before the test, the sample was degassed at 180 °C for 6 h under vacuum conditions. The pore size was obtained by isothermal adsorption branch analysis using the Barret–Joyner–Halenda (BJH) model. Thermogravimetric analysis was performed in a nitrogen atmosphere using GA/DSC1 scanning calorimetry and a thermogravimetric synchrometer (Mettler Toledo, Zurich, Switzerland) at a heating rate of 5 °C/min in the temperature range of 25 °C~800 °C.

### 3.3. Synthesis of the N-CDs/Fe_3_O_4_ Nanocomposite

The preparation of N-CDs/Fe_3_O_4_ involved two steps. The first step was the synthesis of mesoporous Fe_3_O_4_ nanoparticles, and the second step was the in situ synthesis of N-CDs accompanied by loading N-CDs onto mesoporous Fe_3_O_4_ nanoparticles. The mesoporous Fe_3_O_4_ nanoparticles were prepared according to a method reported in the literature [39]. Briefly, FeCl_3_·6H_2_O (1 g) was placed in a beaker containing ethylene glycol (20 mL). A uniformly yellow solution was obtained after stirring for 30 min. Then, 3 g of sodium acetate and 1,2-ethylenediamine (10 mL) were added under magnetic stirring, leading to a brownish solution. The obtained solution was then transferred to a polytetrafluoroethylene autoclave and reacted for 8 h at 200 °C. The black solid obtained from the reaction was thoroughly washed with ultrapure water and ethanol. Then, the mesoporous Fe_3_O_4_ nanomaterials were obtained through magnetic separation.

To in situ synthesize N-CDs, L-histidine (0.1 g) was mixed with the prepared mesoporous Fe_3_O_4_ nanomaterials (10 mg) in a beaker containing 10 mL of ultrapure water. Then, the mixture was transferred to a polytetrafluoroethylene autoclave and reacted at 180 °C for 10 h. After the reaction, a black solid was obtained through magnetic separation. After washing repeatedly with ultrapure water and ethanol, the N-CDs/Fe_3_O_4_ nanozyme was obtained.

### 3.4. Peroxidase-Like Activity of N-CDs/Fe_3_O_4_

The enzymatic properties of the N-CDs/Fe_3_O_4_ magnetic nanozyme were studied using TMB or H_2_O_2_ as substrates. The mixture of TMB (0.5 mM), H_2_O_2_ (6.6 mM), and N-CDs/Fe_3_O_4_ (10 μg/mL) in NaAc–HAc buffer (0.1 M, pH = 4.0) was reacted at room temperature for 10 min. Then, the UV–visible absorption spectrum of the mixed solution was recorded, and the absorbance value at 652 nm was measured as a function of time. For comparison, the solution without the addition of H_2_O_2_ or N-CDs/Fe_3_O_4_ was also investigated. To study the possible synergistic effect between N-CDs and Fe_3_O_4_, N-CDs were synthesized using the same method without the addition of Fe_3_O_4_. To investigate the possible catalytical performance of N-CDs and Fe_3_O_4_, the mixture of TMB (0.5 mM), H_2_O_2_ (6.6 mM), N-CDs (1.3 μg/mL), or Fe_3_O_4_ (8.7 μg/mL) in NaAc–HAc buffer (0.1 M, pH = 4.0) was reacted at room temperature for 10 min. Then, the UV–visible absorption spectrum of the mixed solution was recorded.

The affinity between N-CDs/Fe_3_O_4_ and the enzyme substrates was investigated by measuring the steady-state kinetic parameters. Under the optimal conditions, the concentration of TMB was fixed at 0.5 mM, and the concentration of H_2_O_2_ was changed from 0.1 mM to 0.7 mM. When TMB was used as the substrate, the concentration of H_2_O_2_ was fixed at 0.2 mM, and the concentration of TMB was chosen in the range from 0.1 mM to 0.7 mM. Using the absorbance curve of the system at 652 nm over time, the Michaelis constant (*K*_m_) and the maximum initial reaction rate (*V*_max_) of N-CDs/Fe_3_O_4_ were calculated using the double reciprocal curve of the Michaelis equation.

### 3.5. Determination of the Specific Activity and Recyclability Performance of N-CDs/Fe_3_O_4_

Different concentrations of N-CDs/Fe_3_O_4_ were added to the NaAc–HAc buffer (0.1 M, pH = 4.0) containing H_2_O_2_ (1 M) and TMB (0.5 mM). The obtained solution was incubated for 10 min at 40 °C. Subsequently, the absorbance at 652 nm was recorded as a function of time. The specific activity (SA) was calculated according to the following formula:

b_nanozyme_ = *V*/ε _Δ_*A*/_Δ_T

SA = b_nanozyme_/m

where b_nanozyme_ represents the catalytic activity of the nanozyme; *V* is the total volume of the reaction solution (μL); ε is the molar absorption coefficient (TMB: ε_652nm_ = 39,000 M^−1^ cm^−1^); _Δ_*A*/_Δ_T is the initial rate of change of the absorbance at 652 nm; m is the weight of the nanozyme in each measurement (mg).

The recyclability of N-CDs/Fe_3_O_4_ was achieved by magnetic separation. The activity of the recycled nanozyme was investigated by measuring the absorbance value of the mixed solution at 652 nm. 

### 3.6. Colorimetric Detection of Glucose

The medium for the colorimetric detection of glucose was the NaAc–HAc buffer solution (0.1 M, pH = 4.0) containing TMB (0.5 mM), N-CDs/Fe_3_O_4_ (10 μg/mL), and Gox (0.1 mg/mL). Different concentrations of glucose were added to the above detection medium and incubated at 37 °C for 40 min. Subsequently, the UV–visible absorption spectrum of the mixed solution was recorded. To explore the selectivity of the colorimetric detection of glucose, several common substances and inorganic salt ions in serum were selected as the possible interferences, including Na^+^, Mg^2+^, K^+^, urea, cysteine, glycine, glutamate, ascorbic acid, and dopamine. The detection performance of glucose in the absence or presence of the interferences was measured. The concentration of the possible interfering substances used was 2 mM. To evaluate the accuracy of the sensing of glucose in a real sample, the serum of a healthy woman (provided by Guangxi Medical University Cancer Hospital) was diluted by a factor of 50 before the measurement.

### 3.7. Visual Glucose Detection Based on an Integrated Hydrogel

Visual glucose detection was investigated based on an agarose hydrogel integrated with the nanozyme (N-CDs/Fe_3_O_4_), the biological enzyme (Gox), and TMB. Briefly, agarose (45 mg) was dissolved in 1 mL of boiling water. Then, TMB (1 mL, 5 mM), NaAc–HAc buffer (1 mL, 0.1 M, pH = 4), and N-CDs/Fe_3_O_4_ (0.3 mL, 0.1 mg/mL) were added. After the solution was mixed, it was cooled to 45 °C, and then Gox (0.3mL, 1 mg/mL) was added before shaking well. Then, the solution was added to the mold (96 well plate, 200 μL per well), cooled, and molded to obtain the hydrogel. The integrated hydrogel was obtained after soaking in a 50% ethylene glycol solution for 60 min. For the visual detection of glucose, the integrated hydrogel was added to the solution of glucose at different concentrations and reacted at 40 °C for 30 min. After the reaction, the hydrogel was taken out, and photos were taken with a smartphone. Then, the data for optical primary colors including red (*R*), green (*G*), and blue (*B*) were extracted.

## 4. Conclusions

In this work, a magnetic nanozyme was synthesized using a simple two-step synthesis method by modifying mesoporous Fe_3_O_4_ with N-CDs. The N-CDs/Fe_3_O_4_ nanozyme contains abundant nitrogen and oxygen functional groups on its surface and possesses good peroxidase-like activity and high magnetic susceptibility. The investigation on the nanozyme parameters verified a high substrate specificity. Based on the excellent peroxidase activity of N-CDs/Fe_3_O_4_ and the catalytic oxidation of glucose by glucose oxidase, a colorimetric sensor was constructed to achieve the sensitive detection of glucose. N-CDs/Fe_3_O_4_ can be recycled using magnetic separation and exhibited high reusability. In addition, an integrated hydrogel containing the N-CDs/Fe_3_O_4_ nanozyme, glucose oxidase, and TMB was prepared and exhibited great potential in the visual detection of glucose. When the synthesized magnetic nanozyme was combined with other enzymes such as cholesterol oxidase, a platform for detecting other metabolites can be constructed. Magnetic nanozymes are expected to demonstrate broad application in the colorimetric detection of metabolites.

## Figures and Tables

**Table 1:** Comparison between *K*_m_ and *V*_max_ of a natural enzyme or different nanozymes.

Catalyst	Subtrate	*K*_m_ (mM)	*V*_max_ (10^−8^ M/s)	References
N-CDs/Fe_3_O_4_	H_2_O_2_ TMB	0.719 0.607	1.03 20.5	This work
HRP	H_2_O_2_ TMB	3.7 0.434	8.71 10	[44]
MFNCDs	H_2_O_2_ TMB	0.0044 0.0136	21.17 17.91	[45]
N-CDs	H_2_O_2_ TMB	0.764 0.115	17.2 2.48	[46]
Fe_3_O_4_	H_2_O_2_ TMB	154 0.0980	9.78 3.44	[44]
Fe,N-CDs	H_2_O_2_ TMB	0.350 0.400	1.61 1.19	[47]
Fe-CDs	H_2_O_2_ TMB	97.64 0.348	0.424 0.309	[48]

HRP: horseradish peroxidase; MFNCDs: metal oxide hybrid with nitrogen-doped carbon dots; Fe,N-CDs: Fe- and N-co-doped carbon dots; Fe-CDs: Fe-doped carbon dots.

## Data Availability

The data presented in this study are available on request from the corresponding author.
